# Hexosomes with Undecylenic Acid Efficient against *Candida albicans*

**DOI:** 10.3390/nano8020091

**Published:** 2018-02-07

**Authors:** Marijana Mionić Ebersold, Milica Petrović, Wye-Khay Fong, Debora Bonvin, Heinrich Hofmann, Irena Milošević

**Affiliations:** 1 Powder Technology Laboratory, Institute of Materials, Ecole Polytechnique Fédérale de Lausanne, 1015 Lausanne, Switzerland; petrovicmilica21@gmail.com (M.P.); debora.bonvin@gmail.com (D.B.); heinrich.hofmann@epfl.ch (H.H.); 2 Faculty of Medicine, University of Niš, 18000 Niš, Serbia; 3 Adolphe Merkle Institute, University of Fribourg, 1700 Fribourg, Switzerland; khay.fong@unifr.ch

**Keywords:** undecylenic acid, lipid-based liquid crystal, hexosome, antifungal, fungicidal, anti-*Candida*, *Candida albicans*

## Abstract

Due to the growing issues with fungal infections, especially with *Candida*, there is still a need to develop novel anti-*Candida* materials. One of the known antifungal agents is undecylenic acid (UA), which still cannot be efficiently used due to its oily nature, and thus limited solubility. By taking advantage of the properties of UA, we developed an emulsion with hexagonal phase, i.e., hexosomes, whose structure and morphology was studied by small-angle X-ray scattering and cryo-electron microscopy, respectively. The presence of UA in the hexosome was confirmed by spectroscopy. Moreover, we studied the anti-*Candida* effect of hexosomes and their cytotoxicity toward human cells. The minimal inhibitory concentration for the 50% and 90% *Candida*-growth reduction was found at 0.01 and 0.16 wt % hexosomes, respectively (i.e., 2 and 32 pg_hex_/*C.a.*cell, respectively). The percentage of metabolically active *Candida* was reduced by 72–96% at hexosome concentrations of 1.0–8.2 pg_hex_/*C.a.*cell as compared to untreated *Candida*. Furthermore, at the same concentration range the embedded filamentation test after 24 and 48 h showed the inhibition of both the filamentation and growth of *Candida*, while the preliminary toxicity test showed that hexosomes were nontoxic for human cells. All these render the here-developed hexosomes with UA efficient and promising anti-*Candida* agents.

## 1. Introduction

Fungal infections are recognized as one of the major health issues affecting millions of people worldwide [1,2] and *Candida* is among the four fungal species that pose the largest issue [1]. There are numerous *Candida* species, but *Candida albicans* (*C.a.*) is among those most found in clinical cases [3]. Therefore, numerous studies have been dedicated to combat *C.a.* [3,4,5,6,7,8,9,10,11]. In fact, the current state-of-the-art clinical treatments, which are mainly based on echinocandins and fluconazole, still cannot combat candidiasis, and the mortality from systemic candidiasis still remains ~50% [1]. Among the numerous factors which contribute to the pathogenicity of *C.a.*, the ability to switch between yeast and hyphal growth forms (dimorphism) is recognized as the most critical one [6,12]. Thus, novel anti-*Candida* materials, which would ideally inhibit both the *C.a.* growth and the yeast-to-hyphae transition are urgently need [4,6,9,12,13].

There are numerous drug delivery systems, but lipid-based liquid crystals (LLC) are one of the most promising and, consequently, there is increasing interest in their use and development. LLC are composed of lipid amphiphilic molecules such as monoglycerides, which are known to self-assemble in aqueous environments. Moreover, diffusion-controlled release has been shown to be effective when using reversed bicontinuous cubic or inverse hexagonal phases [14,15]. They are composed of water cylinders enveloped by lipid monolayers and arranged in a two-dimensional hexagonal architecture. In excess of water, LLC can be dispersed into sub-micron sized colloidal particles, maintaining the internal nanostructure of the bulk system [16,17,18,19]. Thus, thermodynamically stable colloidal dispersions of the inverse bicontinuous cubic phases or hexagonal phases, named cubosomes or hexosomes, respectively, can be also produced, making appealing drug delivery systems [14,20,21,22,23]. The colloidal stability of these LLC particles, also known as ISAsomes (Internally Self-Assembled particles), is achieved by the action of stabilizing polymers [17]. One of the most popular stabilizers is probably the synthetic triblock copolymer poloxamer 407, commercially known as F127, consisting of two poly(ethylene oxide) blocks (PEO) separated by a poly(propylene oxide) block (PPO), which is expected to give long-term stability to the system [17,18,24,25]. Moreover, the internal nanostructure of cubosomes or hexosomes has demonstrated their potential in biomedical applications [26,27,28]. Therefore, LLC could be used as nanocarriers with an appropriate antifungal agent. In that case, the antifungal agent would need to have a chemical structure that would allow its incorporation into LLC.

Among the numerous antifungal agents, undecylenic acid (UA) has been known and recognized as a fungicidal substance for decades [29]. Moreover, one recent study revealed the anti-*Candida* mechanisms of action of UA, which reduces the hypha-to-yeast ratio and inhibits: hyphal formation, adhesion, mitochondrial activity, cell proliferation, transcriptional regulation of the cell membrane virulent factors, and biofilm formation [30]. Considering this exceptional potential of UA against *C.a.*, it is of great interest to use UA as an anti-*Candida* agent. For that, the main challenge is to incorporate UA into a carrier in a way that enables UA to be released in an amount sufficient to show an anti-*Candida* effect. Unfortunately, the use of this monounsaturated fatty acid as an antifungal drug is disabled by its oily nature and hence, by its limited solubility [31]. Furthermore, UA is a surfactant, whose chemical nature could be suitable for building LLC.

Here, we developed hexosomes with incorporated UA. Their physicochemical properties, such as hydrodynamic diameter, ζ-potential, chemical nature, and morphology structure, were studied by dynamic light scattering (DLS), Fourier transform infrared spectroscopy (FTIR), cryo-electron microscopy, and small-angle X-ray scattering (SAXS), respectively. As the first evaluation of the anti-*Candida* properties of the hexosomes, we calculated the minimal inhibitory concentration (MIC) for the 50% (MIC50) and 90% (MIC90) reduction of the *C.a*. growth after 24 and 72 h of incubation. Moreover, to evaluate the potential of hexosomes loaded with UA as an anti-*Candida* agent, we determined the percentage of metabolically active *C.a.* cells and their toxicity towards human cells at the same concentrations of hexosome per corresponding cell in both cases. In addition, we investigated the effect of hexosomes on the morphology and growth of *C.a.* cells in embedded conditions after 24 and 48 h of incubation.

## 2. Results and Discussion

### 2.1. Physicochemical Properties of Hexosomes

UA was emulsified to improve the delivery and reduce the toxicity of such a molecule. The nanocarrier formulation was obtained by mixing melted monoglycerides (i.e., Dimodan U/J, DU) with UA and then dispersing them with an ultrasonic tip in an aqueous solution containing Pluronic F127. F127 is well-known for efficiently stabilizing lipid mesophases, even over long-term periods [17]. The hydrophobic moieties (i.e., PPO) of the polymer are adsorbed at the surface of the particles, whereas the hydrophilic ones (i.e., PEO) build a corona that sterically stabilizes the dispersion [32].

The obtained emulsion had a negative surface potential with a ζ of −31 ± 1 mV. Moreover, the emulsions, stored at 4 °C, were stable over one month and the average hydrodynamic diameters, *D*_h_, obtained by three measurements by DLS, were 286 ± 14 nm and 342 ± 50 nm in intensity and volume weighted distributions, respectively (see Supplementary Figure S1).

**Figure 1:**
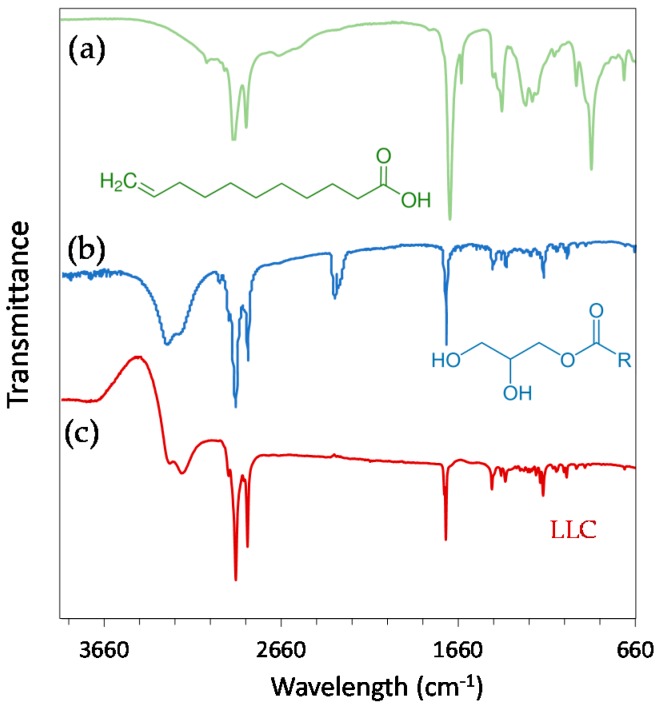
Fourier transform infrared (FTIR) spectra of (**a**) undecylenic acid; (**b**) Dimodan U/J (monoglycerides) and (**c**) lipid-based liquid crystals (LLC). The molecules corresponding to each FTIR spectrum have been added below.

All of the lipid components as well as the final LLC were investigated using FTIR spectroscopy (Figure 1). The region between 1500 and 900 cm^−1^ is often referred to as the fingerprint region, related to both C–O–C stretching and C–H bending [33]. The strong peak in the region at 1700–1800 cm^−1^ in DU and UA spectra can be attributed to carbonyl (C=O) stretching vibration mode (saturated aliphatic esters or carboxylic acid, respectively). In the case of UA, a prominent additional peak is observed at 1640 cm^−1^ and is assigned to C=C vibration mode. This peak is very weak in the case of DU. The strong bands in the region 2800–3000 cm^−1^ may be indicative of symmetrical and asymmetrical stretching of C–H bonds in –CH_3_ and –CH_2_. Finally, the O–H stretch appears as a broad peak between 3000 and 3500 cm^−1^ for both DU and LLC. The analysis of FTIR spectra showed that both UA and DU are present in the final LLC particles. However, since LLC contain 70% of DU and 30% of UA in their nanostructure, the most prominent vibration bands on the spectrum are those of DU.

**Figure 2:**
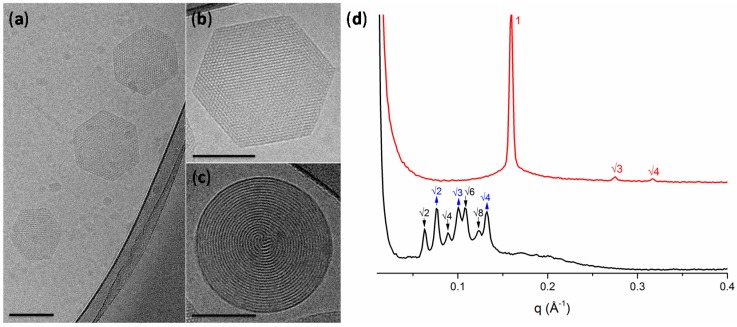
(**a**–**c**) Representative cryo-electron microscopy micrographs of hexosomes (all scale bars represent 100 nm); (**d**) small-angle X-ray scattering profiles of the lipid-based liquid crystals without (black) and with undecylenic acid (red). The cubic phases are indicated by arrows: black, down arrows show the reflections of the Im3m cubic phase; blue, up arrows show the reflections of the Pn3m cubic phase. The reflections are annotated above the Bragg peaks.

Figure 2a–c shows a representative cryo-TEM micrograph of the LLC particles formulation with sizes in agreement with DLS measurements (for the additional TEM micrographs see Supplementary Figure S2). The DU/water system is known to form a Pn3m cubic mesophase in excess of water [19]. However, the molecular packing of the DU (composed of ~60% of monolinolein) can be affected by the hydrocarbon chain space obtained upon the solubilization of an additive component [16,34]. In our case, due to the hydrophobic nature of UA, its addition to DU led to an increase of the negative curvature and to the formation of hexosomes, as observed by cryo-TEM. The hexagonal phase can be clearly identified by the presence of curved striations (Figure 2c) or hexagonal periodicity (Figure 2b) commonly observed in liquid crystalline nanoparticles characterized by reverse hexagonal symmetry.

LLC mesophases are readily identified through SAXS, Figure 2d, where each phase can be identified by its characteristic Bragg peak positions. The H_2_ phase is identified by reflections at 1:√3:√4. These results confirmed that the presence of the UA additive results in the formation of the H_2_ phase with a lattice parameter of 45.6 Å. On the contrary, in the blank dispersion, composed only of DU stabilized by F127, no H_2_ phase was observed. For the double diamond cubic phase (Pn3m), the Bragg reflections occur at relative positions in *q* at √2:√3:√4:√6; while for the primitive bicontinuous cubic phase (Im3m), the Bragg peaks occur at *q* = √2:√4:√6:√8. This mixed Pn3m + Im3m cubic phase has been previously observed in cubosomes formed by the utilized ratio of dimodan + F127 [35]. The lattice parameters of the two cubic phases were found to be *a_(_*_Im3m)_ = 142.3 Å and *a*_(Pn3m)_ = 115.6 Å. The coexistence of these phases approximately follows the Bonnet relation, described as the ratio of the cubic unit cell parameters of the Im3m:Pn3m [36]. In this case, a ratio of 1.231 was obtained, which is comparable with the literature value of 1.279 [36].

### 2.2. Anti-Candida Properties of Hexosomes

Typically, results of the biological tests are expressed as a function of the studied material (here hexosomes) or an active component (here UA). However, even though biological tests are performed with the same concentration of the studied material, the ratio of the studied material and cells are very often different between tests, due to the difference in the various test parameters (such as the volume of the cells’ suspension, concentration of cells, or volume of the studied material). Therefore, comparing results between such tests can be difficult and can lead to misinterpretations. In order to overcome this common issue, we presented results of all here-performed biological tests as a function of the concentration of hexosomes per cell (human cell if not indicated differently in the case of *C.a.* cells). 

**Figure 3:**
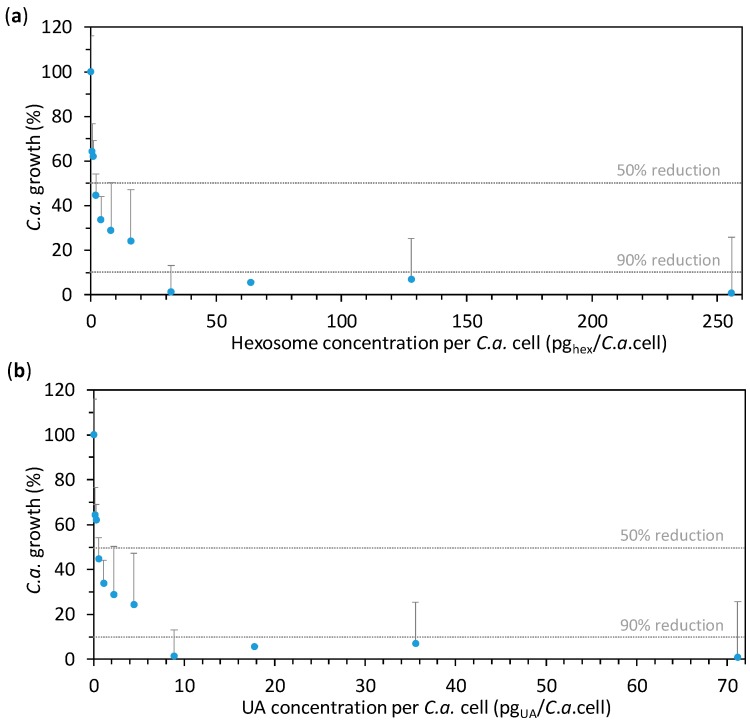
The effect of hexosomes on the *Candida albicans* (*C.a.*) cell growth in the presence of the given concentrations of hexosomes (evaluated by measuring the absorbance at 600 nm, A_600_). The results obtained for *C.a.* cells (concentration of 10^5^ *C.a.*cells/mL) after 24 h of incubation are expressed as average percentages of A_600_ readings compared to the control (0% hexosomes) versus the concentration: (**a**) of hexosomes per *C.a.* cell and (**b**) of undecylenic acid (UA) per *C.a.* cell (all values are given as mean ± standard deviation). The vertical dashed lines indicate 50% and 90% of the *C.a.* growth reduction.

The effect of hexosomes containing UA on the growth of *C.a.* cells was studied for different concentrations of hexosomes prepared in two-fold serial dilution starting from the final hexosome concentration of 1.2784 wt % (corresponding to 0.3559 wt % of UA, 255.7 pg_hex_/*C.a.*cell, or 71.2 pg_UA_/*C.a.*cell). The lowest final hexosome concentration in the seral dilution was 0.0025 wt % (corresponding to 0.0007 wt % of UA, 0.5 pg_hex_/*C.a.*cell, or 0.1 pg_UA_/*C.a.*cell). After 24 h of incubation at 37 °C, the absorbance of the *C.a.* cells’ suspensions containing the given concentrations of hexosomes was measured at 600 nm, A_600_. In order to calculate the percentage of *C.a.* growth, the obtained results were expressed as average percentages of A_600_ readings as compared to the control (0% hexosomes). The so-obtained results are given in Figure 3a as a function of hexosome concentration per *C.a.* cell, while the same results as a function of hexosome (i.e., UA) concentration are given in Supplementary Figure S3. It can be seen that MIC50 was at about 2 pg_hex_/*C.a.*cell, while MIC90 was found at about 32 pg_hex_/*C.a.*cell. Since the dose of UA to which *C.a.* cells are exposed could not be measured, we calculated the maximal theoretical amount of UA to which a *C.a.* cell could be exposed to in a given experiment (if all UA from the hexosomes would come into the suspension of the incubated *C.a.* cells) by dividing the total mass of UA in hexosomes by the number of incubated *C.a.* cells. Therefore, the percentage of *C.a.* growth is also given as a function of the so-obtained UA concentrations per *C.a.* cell (Figure 3b). The corresponding MIC50 and MIC90 are at about 0.6 and 8.9 pg_UA_/*C.a.*cell, respectively. Importantly, the value for MIC90 (8.9 pg_UA_/*C.a.*cell, corresponding to 0.0445 wt % of UA) is much lower than a previously reported value (256 µg_UA_/mL) for *C.a.* cells incubated at 10^7^ *C.a.*cells/mL [37]. This is also lower than MIC90 reported in our previous study, which was found to be between 25 and 50 pg_UA_/*C.a.*cell also at a *C.a.* concentration of 10^7^ *C.a.*cells/mL [38]. This difference could be due to the reported higher efficacy of the nanostructured emulsions as compared to the other drug-delivery systems [14,15], but also due to the different concentrations tested in these studies. Indeed, it is known that an increase of the cells’ concentration increases the drug-resistance of *C.a.* cells [39,40]. These results highlight the high anti-*Candida* effect of our novel hexosomes loaded with UA. In order to assess this effect after a longer period, the measurements were taken not only after 24 h of incubation, but also after 72 h. Interestingly, after 72 h of incubation, the percentage of *C.a.* growth in the presence of various hexosome (i.e., UA) concentrations was almost the same as that after 24 h (Supplementary Figure S4), confirming the efficacy of the hexosomes against *C.a.*

**Figure 4:**
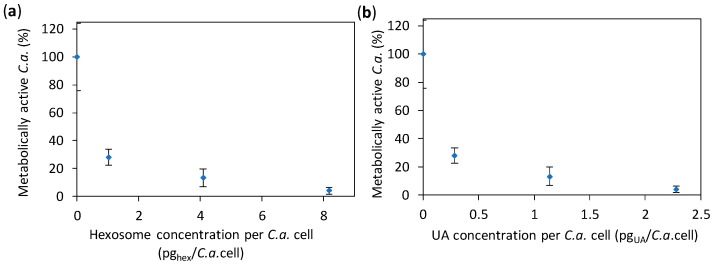
The percentages of metabolically active *Candida albicans* (*C.a.*) cells treated with the different hexosome concentrations were normalized with the number of metabolically active *C.a.* cells treated with media without hexosomes (control). The results are given as a function of: (**a**) the concentration of hexosomes per *C.a.* cell; and (**b**) the concentration of undecylenic acid (UA) per *C.a.* cell; all values are given as the mean ± standard deviation.

Since the results of the previous test showed MIC50 and MIC90 at about 2 and 13 pg_hex_/*C.a.*cell, respectively (corresponding to 0.6 and 8.9 pg_UA_/*C.a.*cell, respectively), we focused the rest of our study on the concentrations in the vicinity of 2 pg_hex_/*C.a.*cell (i.e., 0.6 pg_UA_/*C.a.*cell). We performed an 2,3-bis(2-methoxy-4-nitro-5-sulfo-phenyl)-2H-tetrazolium-5-carboxanilide (XTT) test, which measures the number of metabolically active *C.a.* cells exposed to the hexosome at given concentrations (that are approximately 1.0, 4.1, and 8.2 pg_hex_/*C.a.*cell, i.e., 0.28, 1.14, and 2.28 pg_UA_/*C.a.*cell). The results given in Figure 4 show the relative number of metabolically active *C.a.* cells in the presence of hexosomes (as compared to the control without hexosomes); the same result as a function of hexosome and UA concentrations are given in Supplementary Figure S5. We found that the percentage of metabolically active *C.a.* cells was 28% and 13% at 1.0 and 4.1 pg_hex_/*C.a.*cell, respectively, and only 4% at the highest hexosome concentration. In other words, the number of metabolically active *C.a.* cells was reduced by 72%, 87%, and 96% at the three studied hexosome concentrations, respectively, as compared to the control without hexosomes. The obtained XTT results showed a considerable reduction of the number of metabolically active *C.a.* cells in the presence of hexosomes with UA. Thus, the developed hexosomes showed antifungal activity towards *C.a.* cells at an early stage of colonization (after 24 h). This was so far considered as a substantial issue in the use of UA, which is typically restrained by the concentrations of the released UA, which is insufficient to combat *C.a.*, especially at an early stage of colonization [37]. It is interesting to note that ~0.28 pg_UA_ (more precisely, 0.2847 pg_UA_), corresponding to the amount of UA per *C.a.* cell showing an already substantial anti-*Candida* effect, corresponds to 9.3 × 10^9^ UA molecules, while the estimation of the total number of wall proteins in a *C.a.* yeast cell is 2.9 × 10^6^ proteins [41].

Since the drug-resistance of *C.a.* cells increases along with the cells’ concentration [39,40], the percentage of *C.a.* growth should be higher in the XTT test than in the MIC-determination test for similar drug-to-cell ratios (concentration 10^6^ and 10^5^ *C.a.*cells/mL, respectively). However, in our case, percentages of metabolically active *C.a.* cells in the XTT test were lower than percentages of the *C.a.* growth in the MIC-determination test. This suggests that some of the *C.a.* cells, which contributed to percentages of the *C.a.* growth, were not metabolically active.

**Figure 5:**
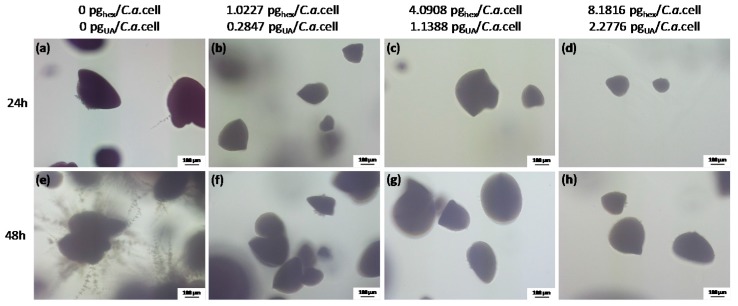
The representative photomicrographs of the *Candida albicans* (*C.a.*) cells imaged after incubation for 24 h (**a**–**d**) and 48 h (**e**–**h**) in the embedded conditions in agar: (**a**,**e**) without hexosomes, which served as a control; (**b**,**f**) with 1.0227 pg_hex_/*C.a.* cell (or 0.2847 pg_UA_/*C.a.*cell); (**c**,**g**) with 4.0908 pg_hex_/*C.a.*cell (or 1.1388 pg_UA_/*C.a*.cell); and (**d**,**h**) with 8.1816 pg_hex_/*C.a.*cell (or 2.2776 pg_UA_/*C.a*.cell); all scale bars represent 100 μm.

In order to gain information on the *C.a.* cell type and the development in the presence of hexosomes at concentrations used in the XTT test as compared to the control without hexosomes, we performed an embedded filamentation assay for both 24 and 48 h of incubation (representative photomicrographs are given in Figure 5). The concentrations of hexosomes in agar were chosen to give the same values of hexosome concentration per *C.a.* cell as in the XTT test. *C.a.* cells in the control sample, i.e., embedded in agar without hexosomes, were, after 24 h of incubation, in the form of spindle-shaped colonies comprising yeast cells with rare peripheral hyphae and/or pseudohyphae, which had lateral yeasts (Figure 5a), as often reported [42,43]. Simultaneously, *C.a.* cells embedded in agar with hexosomes formed yeast colonies with smaller sizes than those in the control and without any hyphae (Figure 5b–d). After 48 h of incubation, control *C.a.* cells embedded in agar without hexosomes formed numerous radially emerging peripheral hyphae, pseudohyphae, and lateral yeasts from spindle-shaped yeast colonies (Figure 5e). Since the incubation was at 37 °C, all embedded colonies formed hyphae, as expected [43]. In contrast, *C.a.* spindle-shaped colonies in agar with hexosomes consisted of yeast with rare yeast outgrowths and without filamentation even after 48 h of incubation (Figure 5f–h). We did not observe large differences between samples with different hexosome concentrations at both timepoints. Overall, these results showed that all studied hexosomes concentrations were sufficient to inhibit the yeast-to-hyphae transition and to suppress *C.a.* growth.

**Figure 6:**
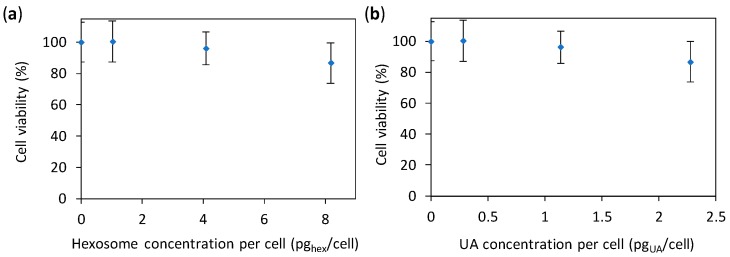
Viability of human A594 cells incubated for 24 h with different concentrations of hexosomes measured with the MTS test. The cell viabilities are given as percentages of viable cells treated with hexosomes (whose absorbance was corrected for the absorbance of hexosomes) normalized with the number of viable cells treated with media without hexosomes (0% hexosomes). The results are given as a function of: (**a**) the concentration of hexosome per cell; and (**b**) the concentration of undecylenic acid (UA) per cells; all values are given as the mean ± standard deviation.

The above given study demonstrates that the developed hexosomes with UA have an anti-*Candida* effect. Nevertheless, to use such antifungal materials in patients, the material need to display an as low as possible toxicity towards human cells. Hence, we performed a preliminary cytotoxicity study (with concentrations of hexosomes adjusted to give the same values per cell as in the previous two tests) by using the common absorbance-based 3-(4,5-dimethylthiazol-2-yl)-5-(3-carboxymethoxypenyl)-2-(4-sulfophenyl)-2H-tetrazolium (MTS) test. However, in such tests the absorbance of the studied nanomaterial (here hexosomes) can contribute to the absorbance of the test itself [44], resulting in unrealistic results (see Supplementary Figure S6). Therefore, we corrected the measured absorbance by subtracting the absorbance of the corresponding amount of hexosomes in the same medium (for details see Materials and Methods). The results (Figure 6) showed a small decrease in cell viability (87%) at the highest concentration of hexosomes per cell (8.2 pg_hex_/cell); the same results as a function of the concentration of hexosome and UA are given in Supplementary Figure S7. Thus, our hexosomes are promising for applications in patients.

So far, UA has been used as a structural agent to build the walls of vesicles, which could be used as nanocontainers [45]. For instance, a recent study exploited the self-assembling behavior of the glycerol monoester of undecylenic acid [46]. Solely concerning UA, Bulut et al. reported the antifungal activity of UA released from emulsions and showed a higher release of UA from emulsions as compared to other liquid phases [47]. However, there were no other reports of UA employed as an antifungal agent in LLC. Beyond LLC, very few studies on materials containing UA for antifungal applications have been reported. For instance, our previous study reported poly(methyl methacrylate) (PMMA) loaded with UA, which showed fungicidal effect against both sessile (attached) and planktonic (free-floating) *C.a*. cells [38]. Besides that, a few studies on one commercial acrylic denture liner (i.e., Coe Soft, GC America, Alsip, IL, USA) reported that it contains UA (70 mM UA [48], or 1–5% UA [49], or a non-specified UA concentration [37]), while according to the producer this product contains zinc undecylenate [50].

Here, a new efficient anti-*Candida* agent was developed in the form of hexosomes loaded with UA. This material simultaneously showed the inhibition of *C.a.* growth and filamentation (i.e., morphogenesis or yeast-to-hyphae transition) at an early stage of colonization (24 h). We also showed that hexosomes were nontoxic to human cells at the studied concentrations, which have been efficient against *C.a.* This is the first reported example of hexosomes with UA, which showed high efficacy against *C.a.*, and of the large potential of LLC nanocarriers for anti-*Candida* applications and, more generally, for anti-fungal applications. 

By taking into consideration global health issues with fungal infections [1,2], the hexosomes reported herein are promising for future use as anti-*Candida* carriers.

## 3. Materials and Methods

### 3.1. Formulation of Hexosomes

Materials used were Dimodan U/J (DU) from DANISCO A/S (Braband, Denmark), which is a monoglyceride mix comprising more than 98 wt % monoglycerides, of which 61.9 wt % are monolinolein (18:2) and 24.9 wt % monoolein (18:1), and undecylenic acid (UA) from Sigma-Aldrich (St. Louis, MO, USA). The emulsifier used was a commercially available triblock copolymer Pluronic F127 (PEO99-PPO67-PEO99, poloxamer 407) from Sigma-Aldrich.

Hexosomes were prepared by a one-pot mixing of 285.9 mg of UA, 668.0 mg of DU, 75.8 mg of F127, and 9 g of deionized water (10 wt % of dispersed material in 90 wt % of water) by applying ultrasound for 15 min using a high intensity ultrasonic tip (Branson Digital Sonifier, Branson Ultrasonic SA, Carouge, Switzerland) at 20% of the maximum power with a 1-s pulse and 1-s pause. No external sample cooling was used. The mixture was allowed to equilibrate at least for 24 h at room temperature before any dilution or biological trials.

### 3.2. Physicochemical Characterisation of Hexosomes

Fourier transform IR (FTIR) spectra of DU, UA, and hexosome suspensions were obtained with a Perkin Elmer Spectrum One spectrometer (series: 69288, Perkin Elmer, Schwerzenbach, Switzerland). Transmittance from 3900 to 650 cm^−1^ were given as the average of eight measured scans for each curve with a resolution of 4.00 cm^−1^.

The zeta potential as well as hydrodynamic diameters (*D_h_*) of the sample were measured at room temperature in zeta potential cuvettes or acrylic cuvettes (Sarstedt, Nümbrecht, Germany), respectively, with a Zetasizer Nano ZS (Malvern Instruments, Worcestershire, UK). Using dynamic light scattering, the reported values were obtained from the average of 3 × 15 measurements after the sample was diluted 1000 times.

For cryo-TEM analysis, an electron microscopy grid (Agar scientific, Essex, UK) with holey carbon film was held in tweezers and 4–5 μL of the sample solution was applied on the grid. The tweezers were mounted in an automatic plunge freezing apparatus (Vitrobot, FEI, Eindhoven, The Netherlands) to control the humidity and temperature. After blotting, the grid was immersed in a small metal container with liquid ethane that was cooled from the outside by liquid nitrogen. The speed of cooling was such that ice crystals do not have time to form. Observation was made at −170 °C in a Tecnai F 20 microscope (FEI, Eindhoven, The Netherlands) operating at 200 kV and equipped with a cryo-specimen holder Gatan 626 (Warrendale, PA, USA). Digital images were recorded with a FalconIII (FEI) camera 4098 × 4098 pixels. Magnification was between 20,000 and 30,000×, using a defocus range of −2 to −3 µm.

The morphology of the dispersed lipid samples was investigated by Small Angle X-ray scattering (SAXS) with a NanoMax-IQ (Rigaku Innovative Technologies, Auburn Hills, MI, USA) at 37 °C. 2D SAXS patterns were radially averaged, yielding 1D SAXS curves of *I*(*q*). The scattering vector, *q*, was calibrated using silver behenate with the *q*-range from 0.06 to 0.6 Å^−1^, where *q* is the length of the scattering vector defined by *q* = 4π/λ sin (θ/2), with *λ* being the wavelength (*λ* = 0.1524 nm) and *θ* being the scattering angle. The dispersed lipid samples were loaded into 2.0-mm quartz capillaries and sealed with epoxy. Measurements were performed at 37 °C. The mean lattice parameter, *a*, was deduced from the corresponding set of observed interplanar distances, *d* (*d* = 2π/*q*), using the appropriate scattering law for the phase structure.

### 3.3. Microorganism and Culture Conditions

In this study, we used *C.a.* ATCC 10231 strain (Microbiologics, LOT 443-518-1, Cat. no. 0443P). *C.a.* stock was kept at −80 °C and, after recovery, kept on Sabouraud 4% Glucose Agar (SGA; Sigma Aldrich 84088) and stored at 4 °C during the experiments. For the determination of the minimum inhibitory concentration and the filamentation assay, the strain was sub-cultured on SGA for 24 h at 37 °C and an inoculum was prepared from freshly grown colonies on SGA at a concentration of 10^5^ *C.a.*cells/mL in 0.9% sterile NaCl (Sodium chloride, 99.5%; Acros, 44730-2500 autoclaved at 121 °C for 20 min). For the XTT assay, an inoculum was adjusted to a concentration of 10^6^ *C.a.* cells/mL in RPMI 1640 medium (Sigma Aldrich, R6504-10x1L).

### 3.4. Anti-Candida Characterization of Hexosomes

#### 3.4.1. Minimum Inhibitory Concentration (MIC)

Two-fold serial dilution of hexosomes was made in RPMI 1640 medium in a 96-well plate and 100 µL of *C.a.* suspension (concentration 10^5^ *C.a.*cells/mL) was added to every well, which gave a final maximal hexosome concentration of 1.2784 wt % (corresponding to 0.3559 wt % of UA, or to 255.68 pg_hex_/*C.a.*cell, or to 71.17 pg_UA_/*C.a.*cell). The last sample in the serial dilution contained a final minimal hexosome concentration of 0.0025 wt % (corresponding to 0.0007 wt % of UA, or to 0.50 pg_hex_/*C.a.*cell, or to 0.14 pg_UA_/*C.a.*cell). Another 96-well plate, which contained the same two-fold dilution of hexosomes in RPMI 1640 medium and 100 µL of medium, was treated under same conditions and served as controls. Both plates were incubated at 37 °C for 24 h and 72 h. Upon incubation, the absorbance the so-obtained suspensions was measured at 600 nm, using a microplate reader (TECAN Infinite M200, Tecan, Männedorf, Switzerland). From the obtained absorbance values, the percentages of viable *C.a.* cells were calculated with the following equation:

percentage of viable *C.a.* cells (%) = [*Abs*(*C.a*.; Hex; medium) − *Abs*(Hex; medium)]/[*Abs*(*C.a*.; medium) − *Abs*(medium)]·100% (1)

where *Abs*(*C.a*.; Hex; medium), *Abs*(Hex; medium), *Abs*(*C.a*.; medium), and *Abs*(medium) denote the absorbance of liquid with *C.a.* and hexosomes and medium, with hexosomes and medium, with *C.a.* and medium, and only with RPMI medium, respectively.

#### 3.4.2. XTT Assay

The preparation of XTT/menadione solution was conducted as follows. For the XTT reduction assay, the XTT (Cayman, CAS 111072-31-2,)-saturated solution at 0.5 g/L was prepared in sterile PBS (Dulbecco’s Phosphate Buffered Saline, Zen-Bio, Inc., Research Triangle Park, NC, USA, DPBS-1000), sterilized by filtration using a 0.22 µm pore-size filter, aliquoted into working volumes, and stored at −20 °C when not used. The stock of the XTT solution was thawed before every assay, and the menadione (Cayman Chemicals, Hamburg, Germany, CAY15950-25g) solution previously prepared in the acetone as a 10 mM stock solution was added to the XTT to have a final menadione concentration of 1 µM. The so-obtained solution is referred to as XTT/menadione.

In the wells of 96-well plates, we first added 100 µL of a *C.a*. inoculum (at a concentration of 10^6^ *C.a.* cells/mL in RPMI 1640 medium) and then suitable amounts of hexosomes suspension to reach the final concentrations of 0.093, 0.292, and 0.454 wt %, corresponding to the concentrations of UA of 0.026, 0.081, and 0.127 wt %, the concentrations of hexosomes per *C.a.* cell of 1.0227, 4.0908, and 8.1816 pg_hex_/*C.a.cell*, and the concentrations of UA per *C.a.* cell of 0.2847, 1.1388, and 2.2776 pg_UA_/*C.a.*cell. The same concentrations of hexosomes in the same volumes of RPMI 1640 medium without *C.a.* served as controls, in addition to RPMI 1640 medium only and *C.a.* cell suspensions in RPMI 1640 medium. The so-prepared well plates were covered with a sterile seal plate film, covered with a lid, sealed with parafilm, and incubated for 24 h at 37 °C. After 24 h of incubation, 100 μL of XTT/menadione was added to every well. The plates were covered with a lid, sealed with parafilm, wrapped with aluminum foil, and incubated in the dark for 3 h at 37 °C. Afterwards, the absorbance was measured with a microtiter plate reader (TECAN Infinite M200, Tecan, Männedorf, Switzerland) at 490 nm. From the obtained absorbance values, the percentage of metabolically active *C.a.* cells (%) was calculated with the following equation:

percentage of metabolically active *C.a.* cells (%) = [*Abs*(*C.a*.; Hex; medium) − *Abs*(Hex; medium)]/[*Abs*(*C.a*.; medium) − *Abs*(medium)]·100% (2)

where *Abs*(*C.a*.; Hex; medium), *Abs*(Hex; medium), *Abs*(*C.a*.; medium), and *Abs*(medium) denote the absorbance of the liquid containing XTT: with *C.a.* and hexosomes and medium, with hexosomes and medium, with *C.a.* and medium, and only with RPMI medium, respectively.

#### 3.4.3. Embedded Filamentation Assay

Hexosome suspensions in YPD-agar were prepared according to a previously described protocol [38] in order to form three different concentrations of hexosomes per *C.a.* cell, which corresponds to the concentrations in the XTT assay: 1.0227, 4.0908, and 8.1816 pg_hex_/*C.a.*cell, and to the concentrations of UA per *C.a.* cell of 0.2847, 1.1388, and 2.2776 pg_UA_/*C.a.*cell. Briefly, after autoclaving and natural cooling down to approximately 40 °C, 5 mL of YPD-agar was added into 50-mL polypropylene flat falcon tubes (Falcon 62.559.001) containing a suitable amount of hexosome suspension. To 5 mL of the so-prepared hexosome suspensions, 100 µL of *C.a.* (concentration 10^6^ *C.a.*cells/mL) was added, mixed, and poured into sterile Petri dishes (with a diameter of 30 mm). After natural cooling down, Petri dishes were incubated at 37 °C and studied by an optical microscope (Nikon Eclipse Ti-E inverted microscope, Nikon Instruments Europe BV, Amsterdam, The Netherlands) after 24 h and 48 h. Photomicrographs were taken through the agar matrix. 

### 3.5. Cytotoxicity Study of Hexosomes

Human A549 cells were cultured in RPMI-1640 medium (Sigma-Aldrich) supplemented with 10% fetal bovine serum and 2% 5000 U·mL^−1^ penicillin, 5 mg·mL^−1^ Streptomycin and 10 mg·mL^−1^ neomycin (Sigma-Aldrich). 4000 A549 cells per well were cultured in 96-well plates at 37 °C for 24 h, and afterwards exposed for an additional 24 h to 100 μL media containing suitable amounts of hexosomes. The same amounts of hexosomes in 100 μL media without cells, as well as cells treated only with medium served as controls. After 24 h of incubation, the supernatant of each well was removed. Then, 100 μL of MTS solution (CellTiter 96^®^ AQueous One Solution Cell Proliferation Assay from Promega, Madison, WI, USA, diluted six times in medium) was added to the cells. After 2 h of incubation in the dark, the absorbance of the formazan product was measured with a microplate reader (Tecan Infinite M200, Tecan, Männedorf, Switzerland) at a wavelength of 490 nm. All experiments were performed in four repetitions. Results are given as means (with standard deviations) of the values obtained in these four repetitions. From the obtained absorbance values, the percentage of cell viability (%) was calculated with the following equation:

percentage of cell viability (%) = [*Abs*(cells; Hex; medium) − *Abs*(Hex; medium)]/[*Abs*(cells; medium) − *Abs*(medium)]·100% (3)

where *Abs*(cells; Hex; medium), *Abs*(Hex; medium), *Abs*(cells; medium), and *Abs*(medium) denote the absorbance of liquid containing MTS: with cells and hexosomes and medium, with hexosomes and medium, with cells and medium, and only with medium, respectively.

## Supplementary Materials

The following are available online at http://www.mdpi.com/2079-4991/8/2/91/s1, Figure S1: The average hydrodynamic diameters; Figure S2: The additional transmission electron microscopy micrographs of hexosomes, Figure S3: The effect of hexosomes on the *Candida albicans* (*C.a.*) cell growth, Figure S4: The effect of hexosomes on the *Candida albicans* (*C.a.*) cell growth, Figure S5: The percentages of metabolically active *Candida albicans* (*C.a.*) cells, Figure S6: Viability of human A594 cells incubated for 24 h with different concentrations of hexosomes, Figure S7: Viability of human A594 cells incubated for 24 h with different concentrations of hexosomes.
