# Supplementary material for: Hexosomes with Undecylenic Acid Efficient against Candida albicans
Source: Nanomaterials (Basel). 2018 Feb 7;8(2):91. doi: 10.3390/nano8020091 (PMC5853723; doi:10.3390/nano8020091)
Supplement: Supplementary file 1 [file nanomaterials-08-00091-s001.pdf]

nanomaterials

MDPI

Supplementary Material

Hexosomes with Undecylenic Acid Efficient against *Candida Albicans*

Marijana Mionić Ebersold [1,*], Milica Petrović [1,2], Wye-Khay Fong [3], Debora Bonvin [1], Heinrich Hofmann [1] and Irena Milošević [1,*]

[1] Powder Technology Laboratory, Institute of Materials, Ecole Polytechnique Fédérale de Lausanne, 1015 Lausanne, Switzerland; petrovicmilica21@gmail.com (M.P.); debora.bonvin@gmail.com (D.B.); heinrich.hofmann@epfl.ch (H.H.)
[2] Faculty of Medicine, University of Niš, 18000 Niš, Serbia
[3] Adolphe Merkle Institute, University of Fribourg, 1700 Fribourg, Switzerland; khay.fong@unifr.ch
* Correspondence: marijanamionic@gmail.com (M.M.E.); irena.markovic@epfl.ch (I.M.); Tel.: +41-76-238-1669 (M.M.E.); +41-21-693-5107 (I.M.)

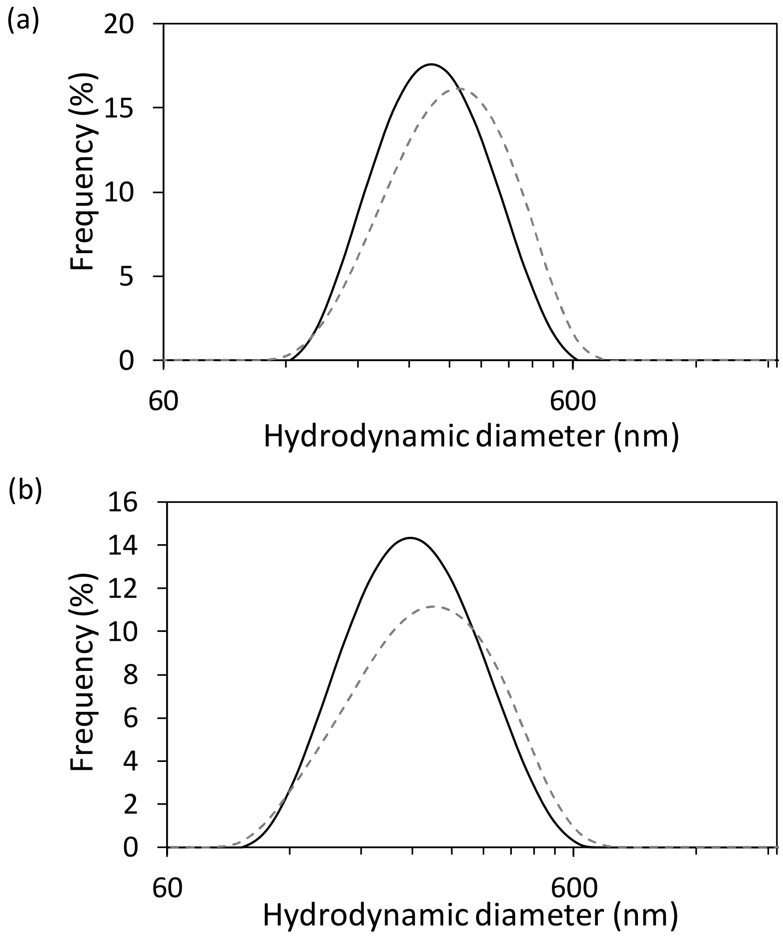

Figure S1. The hydrodynamic diameter, D_h, in water (**a**) and in a saline medium (0.9% NaCl) (**b**) in intensity (solid lines) and volume (dash lines) weighted distributions obtained by dynamic light scattering.

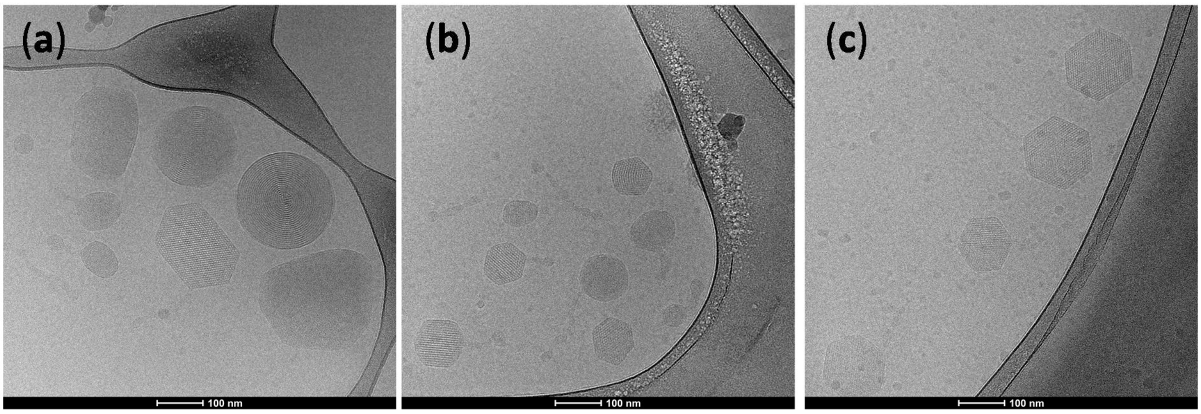

Figure S2. (**a**–**c**) The additional transmission electron microscopy micrographs of hexosomes.

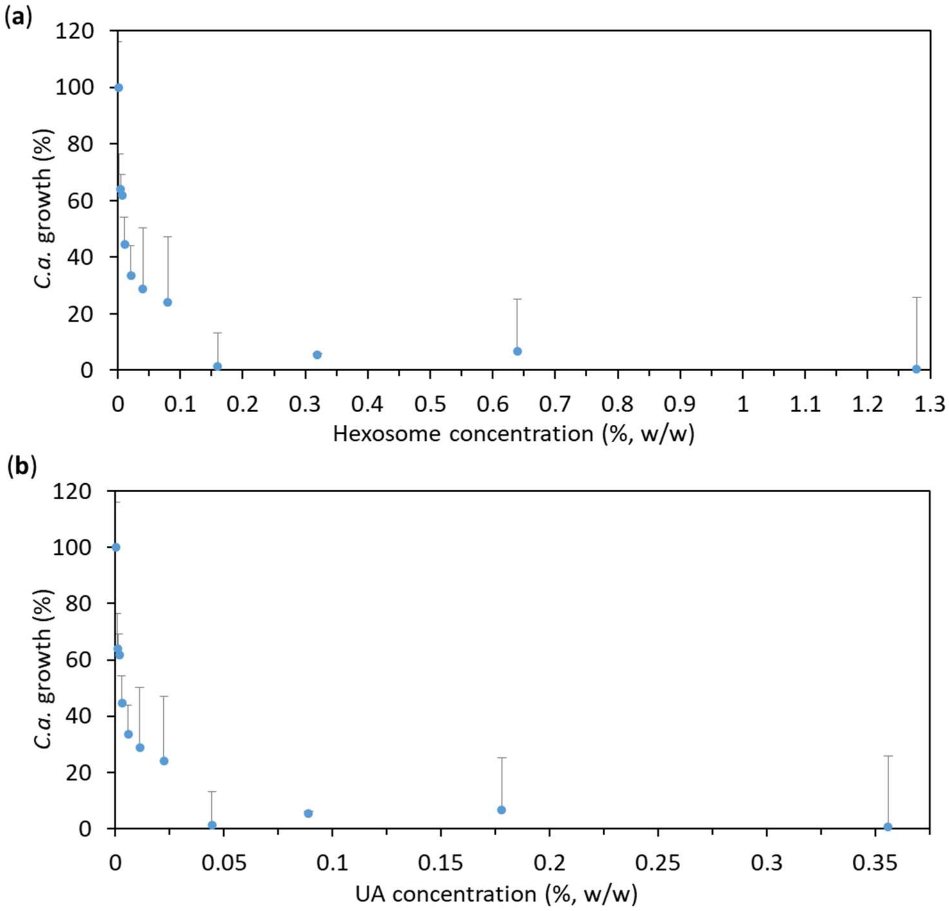

Figure S3. The effect of hexosomes on the *Candida albicans* (*C.a.*) cell growth in the presence of the given concentrations of hexosomes (evaluated by measuring the absorbance at 600 nm, A_{600}). The results obtained for *C.a.* cells (concentration of 10^5 *C.a.*cells/ml) after 24 h incubation are expressed as average percentages of A_{600} readings compared to control (0% hexosomes) *versus* concentration: (**a**) of hexosomes and (**b**) of undecylenic acid (UA) (all values are given as mean ± standard deviation).

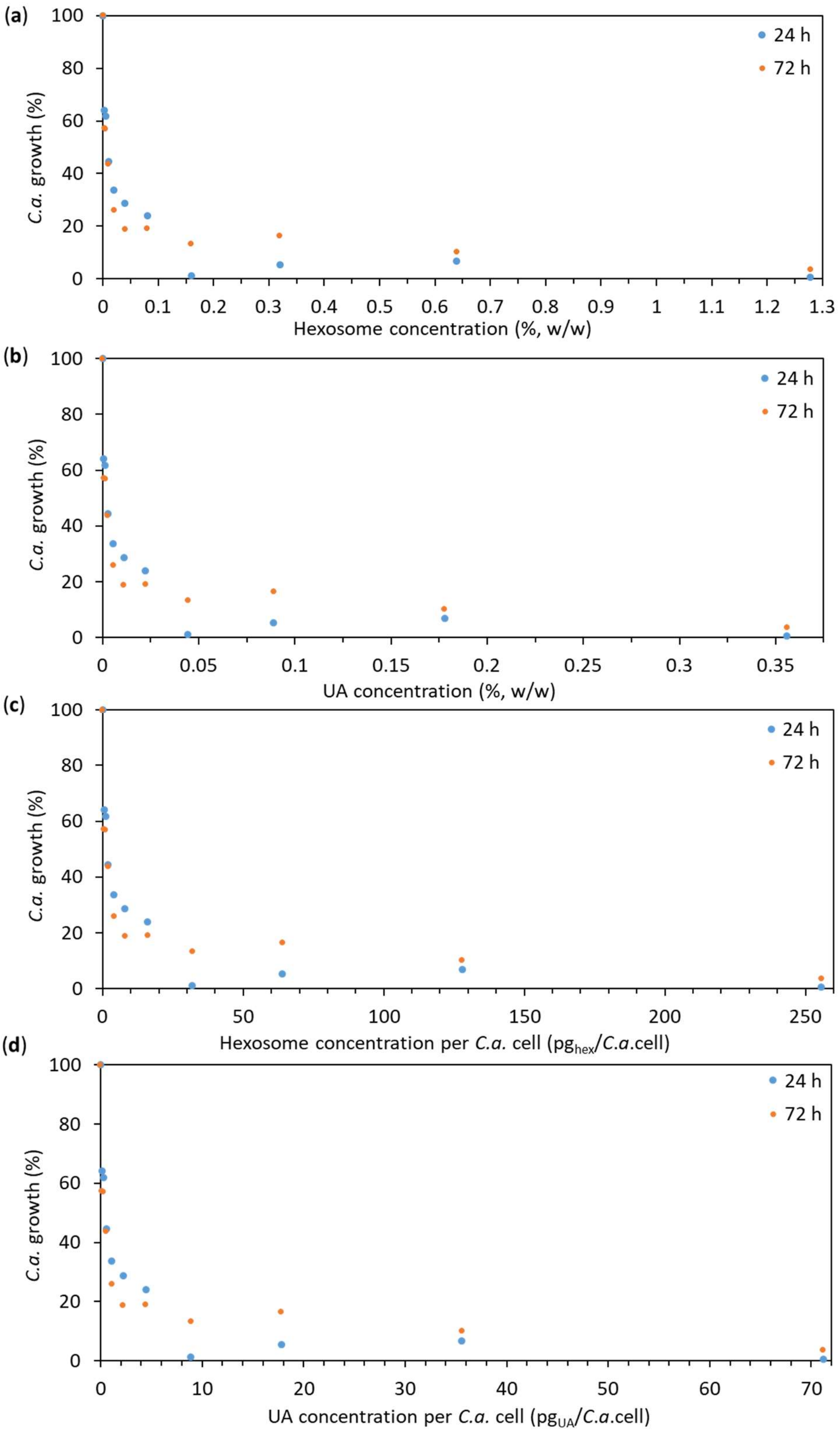

Figure S4. The effect of hexosomes on the *Candida albicans* (*C.a.*) cell growth in the presence of the given concentrations of hexosomes (evaluated by measuring the absorbance at 600 nm, A_{600}). The results obtained for *C.a.* cells (concentration of 10^5 *C.a.*cells/ml) after 24 h and 72 h of incubation are expressed as average percentages of A_{600} readings compared to control (0% hexosomes) *versus* concentration: (**a**) of hexosomes, (**b**) of undecylenic acid (UA), (**c**) of hexosomes per *C.a.* cell, and (**d**) of undecylenic acid (UA) per *C.a.* cell (all values are given as mean ± standard deviation).

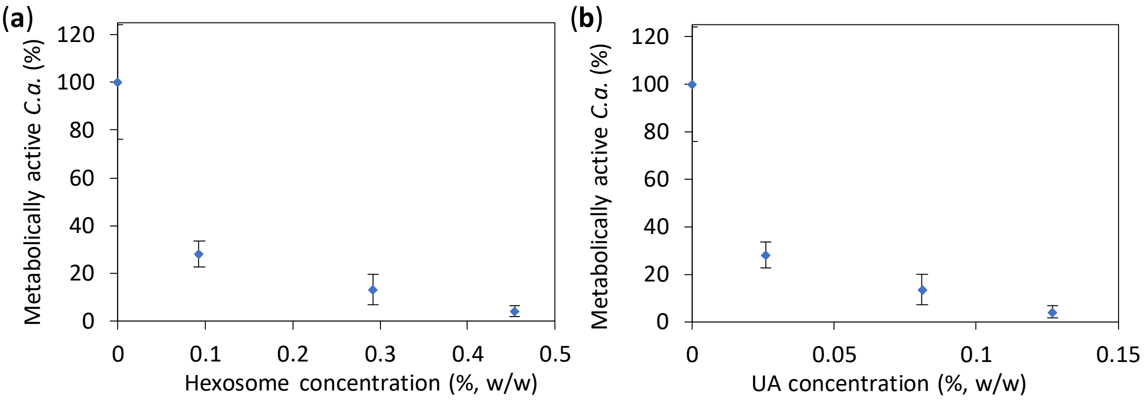

Figure S5. The percentages of metabolically active *Candida albicans* (*C.a.*) cells treated with the different hexosome concentrations were normalized with the number of metabolically active *C.a.* cells treated with media without hexosomes. The results are given as a function of: (**a**) the concentration of hexosome, and (**b**) the concentration of undecylenic acid (UA); all values are given as mean ± standard deviation.

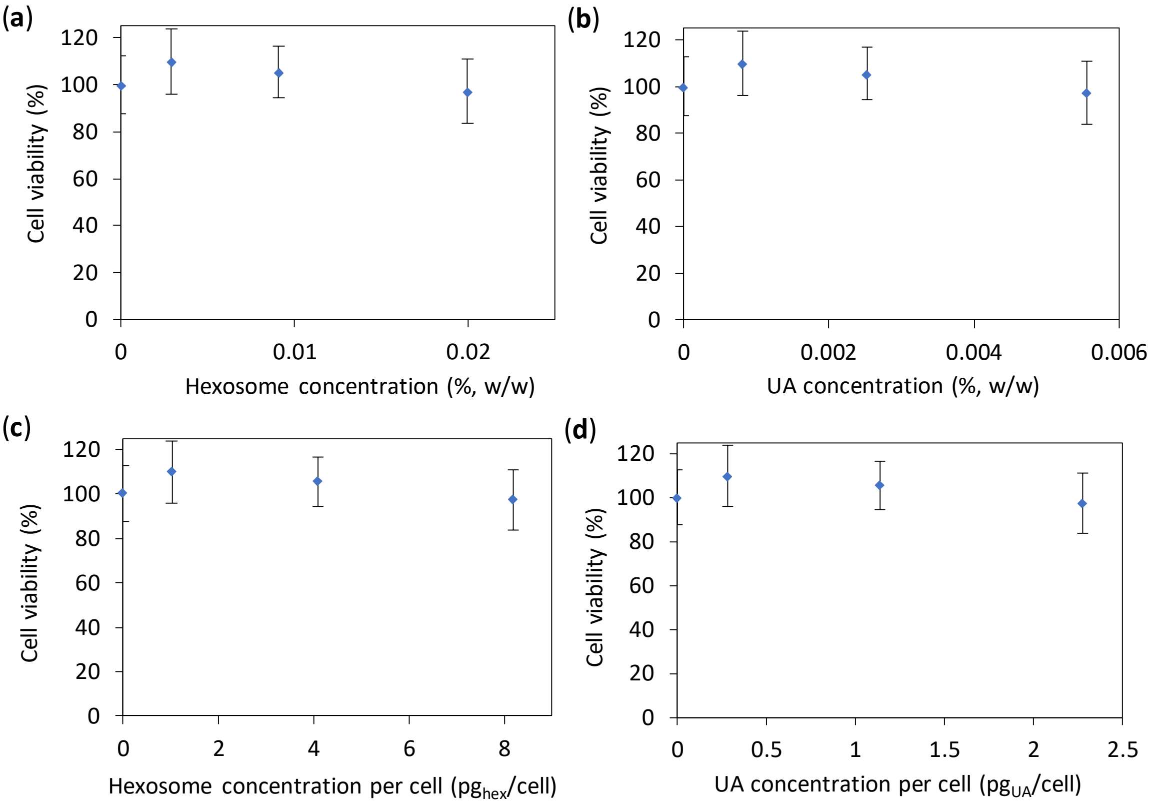

Figure S6. Viability of human A594 cells incubated for 24 h with different concentrations of hexosomes measured with the MTS test. The cell viabilities are given as percentages of viable cells treated with hexosomes normalized with the number of viable cells treated with media without hexosomes (0% hexosomes). The results are given as a function of: (**a**) the concentration of hexosome per cell, and (**b**) the concentration of undecylenic acid (UA) per cells; all values are given as mean ± standard deviation.

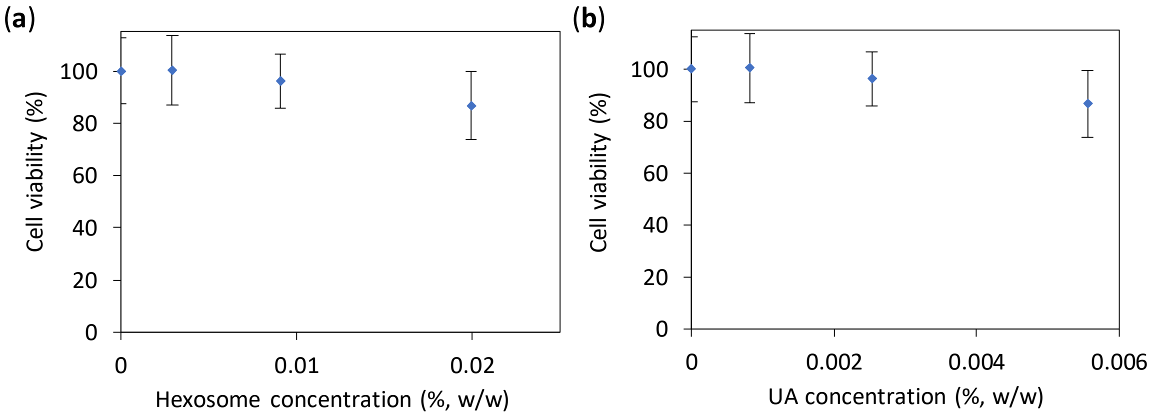

Figure S7. Viability of human A594 cells incubated for 24 h with different concentrations of hexosomes measured with the MTS test. The cell viabilities are given as percentages of viable cells treated with hexosomes (whose absorbance was corrected for the absorbance of hexosomes) normalized with the number of viable cells treated with media without hexosomes (0% hexosomes). The results are given as a function of: (**a**) the concentration of hexosome, and (**b**) the concentration of undecylenic acid (UA); all values are given as mean ± standard deviation.
